# *Schistosoma mansoni* Tegument (Smteg) Induces IL-10 and Modulates Experimental Airway Inflammation

**DOI:** 10.1371/journal.pone.0160118

**Published:** 2016-07-25

**Authors:** Fábio Vitarelli Marinho, Clarice Carvalho Alves, Sara C. de Souza, Cintia M. G. da Silva, Geovanni D. Cassali, Sergio C. Oliveira, Lucila G. G. Pacifico, Cristina T. Fonseca

**Affiliations:** 1 Laboratório de Imunologia de Doenças Infecciosas, Departamento de Bioquímica e Imunologia, Universidade Federal de Minas Gerais, Belo Horizonte-MG, Brazil; 2 Laboratório de Biologia e Imunologia Parasitária, Centro de Pesquisas René Rachou, Fundação Oswaldo Cruz, Belo Horizonte-MG, Brazil; 3 Laboratório de Patologia, Departamento de Patologia, Universidade Federal de Minas Gerais, Belo Horizonte-MG, Brazil; 4 Instituto Nacional de Ciência e Tecnologia em Doenças Tropicais (INCT-DT), CNPq MCT, Salvador-BA, Brazil; Universidade Federal do Rio de Janeiro, BRAZIL

## Abstract

**Background:**

Previous studies have demonstrated that *S*. *mansoni* infection and inoculation of the parasite eggs and antigens are able to modulate airways inflammation induced by OVA in mice. This modulation was associated to an enhanced production of interleukin-10 and to an increased number of regulatory T cells. The *S*. *mansoni* schistosomulum is the first stage to come into contact with the host immune system and its tegument represents the host-parasite interface. The schistosomula tegument (Smteg) has never been studied in the context of modulation of inflammatory disorders, although immune evasion mechanisms take place in this phase of infection to guarantee the persistence of the parasite in the host.

**Methodology and Principal Findings:**

The aim of this study was to evaluate the Smteg ability to modulate inflammation in an experimental airway inflammation model induced by OVA and to characterize the immune factors involved in this modulation. To achieve the objective, BALB/c mice were sensitized with ovalbumin (OVA) and then challenged with OVA aerosol after Smteg intraperitoneal inoculation. Protein extravasation and inflammatory cells were assessed in bronchoalveolar lavage and IgE levels were measured in serum. Additionally, lungs were excised for histopathological analyses, cytokine measurement and characterization of the cell populations. Inoculation with Smteg led to a reduction in the protein levels in bronchoalveolar lavage (BAL) and eosinophils in both BAL and lung tissue. In the lung tissue there was a reduction in inflammatory cells and collagen deposition as well as in IL-5, IL-13, IL-25 and CCL11 levels. Additionally, a decrease in specific anti-OVA IgE levels was observed. The reduction observed in these inflammatory parameters was associated with increased levels of IL-10 in lung tissues. Furthermore, Smteg/asthma mice showed high percentage of CD11b^+^F4/80^+^IL-10^+^ and CD11c^+^CD11b^+^IL-10^+^ cells in lungs.

**Conclusion:**

Taken together, these findings demonstrate that *S*. *mansoni* schistosomula tegument can modulates experimental airway inflammation.

## Introduction

Asthma is characterized by chronic inflammation of the airways and lungs with marked Th2 response, as showed by high concentrations of interleukin (IL)-4, IL-5 and IL-13, IgE production, mucus and eosinophils influx to airways [1]. It is a global health problem that affects people of all ages worldwide and its prevalence is increasing in several countries, especially among children. It is the commonest cause of medical admission in childhood and has a major impact on hospital services for adults [2–5]. The allergic diseases treatment is based on the use of corticosteroids, humanized anti IgE antibody (omalizumab^®^) and antihistamines medications. However, corticosteroids do not cure the pathology, and during extended use, it can cause systemic side effects as easy bruising and bone loss [6–8]. Moreover, omalizumab is used as a treatment in severely allergic asthmatics to reduce inhaled corticosteroid [9] and still adverse effects are observed [10]. Therefore, the search for news molecules for asthma prevention and/or treatment is required.

Some studies support that allergic diseases are suppressed by helminthic infection once helminthes are important modulators of immunity [11–12]. Concerning schistosomiasis, there is a negative association between the infection and allergic episodes, as in endemic areas is observed a low prevalence of allergic asthma [11, 13]. It has been described that a modulatory network with regulatory cells [14–16] and molecules such as IL-10 and TGF-β [1, 17–20] are important factors for protection against allergy. In experimental models of ovalbumin (OVA) induced allergy, several compounds with potential to modulate airway inflammation such as parasite eggs and recombinant proteins were identified in *S*. *mansoni* [21–22]. Using this OVA-induced airway inflammation model, our group has demonstrated the role of Treg cells and IL-10 in modulating inflammatory responses [18, 21–22].

The *S*. *mansoni* tegument is the parasite layer that interacts with the host and it is involved in several features as nutrition, excretion, osmoregulation, sensorial reception, signal transduction, evasion and immune response modulation [23–24]. The *S*. *mansoni* schistosomula tegument (Smteg) is an antigen preparation that has been previously demonstrated by our group to induce increased production of IL-10 by spleen cells and bone marrow derivate dendritic cells [25]. This regulatory property could serve as an important tool to be used against inflammatory diseases such as allergic airway inflammation.

In this study, we demonstrated the ability of Smteg to modulate the experimental airway inflammation induced by OVA, downregulating inflammatory parameters such as number of eosinophils, proinflammatory cytokines, specific anti-OVA IgE and lung pathology. The modulation was associated with increased percentage of CD11b^+^F4/80^+^IL-10^+^ and CD11c^+^CD11b^+^IL-10^+^ cells and IL-10 levels in lungs. These findings are significant not only on the search for new modulatory molecules against airway inflammation, but also an important step toward understanding immune evasion mechanisms used by schistosomes to persist in the definitive host.

## Materials and Methods

### Mice and Smteg preparation

Female BALB/c mice, 6–8 weeks old, were obtained from the Federal University of Minas Gerais (UFMG) animal facility. Mice were housed in cages with a maximum number of 5 animals/cage. The animals had free access to water and food and were monitored every other day. No animal died before the end of the protocol and it was not necessary to apply a protocol for early endpoint. The euthanasia was performed by lethal anesthesia using 500 micro liters of a solution containing 0.002 g of Xilazine and 0.01g of Ketamine injected intraperitoneally. Smteg was prepared as described by Durães et al. (2009) [26], using cercariae from the LE strain obtained from the snails from Centro de Pesquisas René Rachou- CPqRR-Fiocruz (MG-Brazil). Briefly, Cercariae from *S*. *mansoni* were mechanically transformed into skin-stage schistosomula according to Ramalho-Pinto et al [27]. The tegument was removed with CaCl_2_ 0.3M by vortex agitation. The tegument was separated from denuded bodies by centrifugation at 900 *g* for 1 min. The supernatants were pooled and centrifuged at 50000 *g* for 1h at 4°C. The pellet was dialyzed against 1,7% saline for 48 h and physiological saline for 24 h.

### Sensitization, Smteg inoculation and challenge with OVA

**Fig 1:**
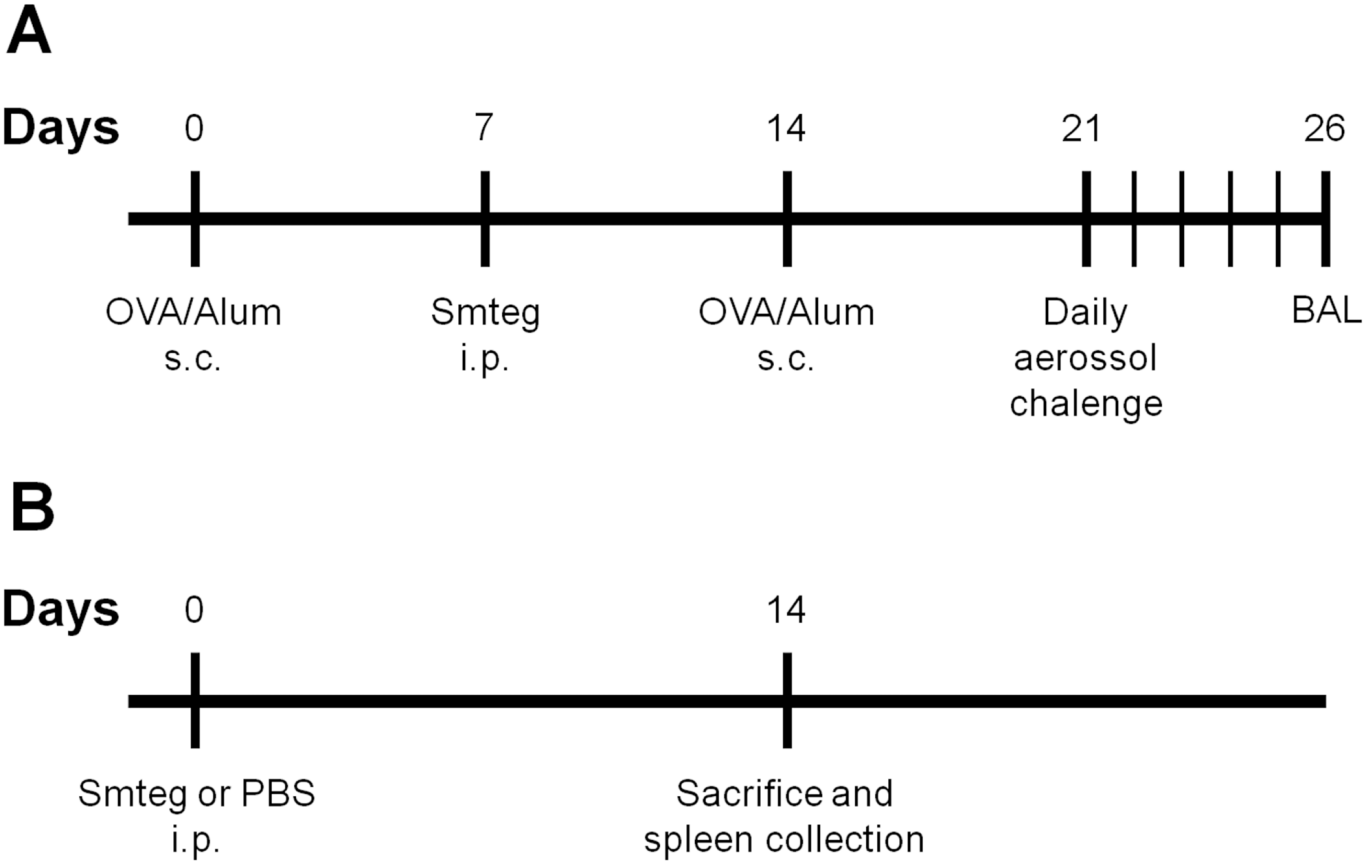
Induction of airway inflammation and inoculation of Smteg in murine model. (**A**) BALB/c mice were sensitized with OVA on days 0 and 14 and received Smteg on day 7. Mice were challenged with aerosol from days 21 to 25 and euthanized on day 26. (**B**) Mice received Smteg or PBS and were sacrificed after 14 days. s.c.–subcutaneous, i.p.–intraperitoneal, BAL- bronchoalveolar lavage.

Airway inflammation was induced in mice as previously described [21]. Briefly, mice (n = 5) were grouped according to the following treatment: PBS (phosphate-buffered saline (PBS)-challenged), Asthma (OVA-challenged) and Smteg/Asthma (inoculated with Smteg and OVA-challenged). All animals were sensitized with OVA twice (Sigma-Aldrich, St Louis, MO, USA; 10 μg in 1 mg of alum), at days 0 and 14. Seven days after the first sensitization, mice of the Smteg/Asthma group received 25 μg of Smteg intraperitoneally. Then, during days 21^st^ to 25^th^ mice were challenged with aerosolized PBS or a solution of OVA 1% in PBS. Twenty-four hours after the last challenge all mice were euthanised by lethal anesthesia (Fig 1A).

Additionally, mice not sensitized with OVA, received 25 μg of Smteg (Smteg group n = 5) or 200 μL of PBS intraperitoneally (Non-treated (NT) group n = 5). Fourteen days after inoculation, mice were euthanised and had the spleen collected in individual basis to access the cytokine profile induce by Smteg inoculation (Fig 1B).

### Bronchoalveolar lavage (BAL)

The tracheas of lethally anesthetized mice were cannulated and the airway lumen was washed twice (first with 500 μl and then with 1 mL) of PBS. The recovered fluids were centrifuged, and cell pellets were ressuspended in 100 μl of Bovine Serum Albumine (BSA) 3%. Total leukocytes were counted using a haemocytometer. Cytospin slides were made and stained with Panótico Rápido^®^ method using triarylmethane, xanthene and thiazin (Laborclin Ltda, Pinhais, PR, Brazil) to determine the cell counts as previously demonstrated [21].

### Measurement of protein extravasation in BAL

The measurement of protein extravasation due to asthma induction was performed using Bradford kit (BioRad, Hercules, CA, USA) according to manufacturer’s instruction. Standard protein dilutions were prepared using BSA (2 mg/mL) in duplicate at concentrations ranging from 0.05 mg/mL to 1.5 mg/mL. Triplicate of BAL samples from animals of each group was placed in 96-well microtiters plates (Nunc) and Bradford reagent was added. After incubation, the plate was read at 595 nm using ELISA reader (BioRad).

### Measurement of IL-5, IL-10, IL-13 and CCL11 levels in lungs

The lung tissue (100 mg) of each animal was homogenized in 1 ml of PBS containing antiproteases (0.1 mM PMSF, 0.1 mM benzethonium chloride, 10 mM EDTA and 20 KI aprotinin A) and 0.05% Tween 20. The samples were then centrifuged for 10 min at 3000*g* and the supernatant was immediately used to detect IL-5, IL-10, IL-13 and CCL11. The cytokines and the chemokine concentrations were measured in lungs of mice using commercially available kits (eBiosciences, San Jose, CA, USA for IL-5, IL-10 and IL-13; R&D Diagnostics, Minneapolis, MN, USA for CCL11) according to the manufacturer’s instructions.

### Measurement of anti-OVA specific IgE antibodies

The measurement of anti-OVA specific IgE antibodies was performed using ELISA. Briefly, Maxisorp 96-well microtiters plates (Nunc) were coated with ovalbumin 10 μg/ml in carbonate-bicarbonate buffer, pH 9.6, for 12–16 hours at 4°C. Then the plates were blocked for 24 hours at 4°C with 100 μl/well of PBS plus 0.05% Tween 20 (PBST)-casein (3%). One hundred microliters of each serum diluted in PBST 1:100 were added per well and incubated for 24 hours at 4°C. Next, samples were incubated with 100 μl/well of anti-IgE (2 μg/mL) at room temperature for 1 hour. Plate-bound antibody was detected by streptavidine-HRP (1:1200) 100 μl/well for 30 minutes at room temperature. Color reaction was developed by addition of 100 μl/well of TMB (Microwell Peroxidase Substrate System from Invitrogen, Camarillo, CA, USA) for 10 minutes and stopped with 50 μl of 5% sulfuric acid per well. The plates were read at 450 nm in an ELISA reader (BioRad).

### Measurement of IL-4, IFN-γ, IL-17 and IL-10 levels in spleen cells culture

Cells that were obtained from the spleens of animals from NT or Smteg groups were washed with saline and the erythrocytes were lysed with a hemolytic solution (155 mM NH_4_Cl, 10 mM KHCO_3_, pH 7.2). Splenocytes were seeded at 10^6^/well into 96-well plates with RPMI 1640 (Gibco, Carlsbad, CA, USA) that was supplemented with 2 mM L-glutamine, 25 mM HEPES, 10% heat-inactivated FBS (Gibco), penicillin G sodium (100 U/ml), and streptomycin sulfate (100 μg/ml). The cells were stimulated with Smteg (25 μg/ml) or concanavalin A (ConA; 5 μg/mL). Unstimulated cells were used as negative controls. After 72 hrs of culture at 37°C, cells supernatants were collected and cytokines levels were measured by CBA Mouse Th1/Th2/Th17 Cytokine Kit (BD, Franklin Lakes, New Jersey, USA) according to the manufacturer’s instructions.

### Lung Pathology

Lungs were collected 24 hours after the aerosol challenge and fixed in 10% buffered formalin. The fragments were then dehydrated, cleared and embedded in paraffin. Serial sagittal sections of the whole lung were cut (3–4 μm thick), stained with Haematoxilin-Eosin (HE) or Gomori Trichrome and examined for cell infiltration as previously demonstrated [28]. For quantitative analysis of collagen deposition, images of the lung sections stained with Gomori Trichrome were captured with a digital camera (Axiocam MRc) connected to a microscope (AxioObserver, Carl Zeiss) using a 10X objective. Collagen deposition (Green area) was measured using Axiovision Release 4.8 software. Images covering all of the lung area from each animal were captured and analyzed. Fibrosis areas were determined and divided by the total area of lung section analyzed in each animal. Results are expressed as fibrosis area (μm^2^)/mm^2^ of lung tissue +/- SD. The number of eosinophils in the lung was determined in HE stained section of 5 animals per group. Images (at least 40 images per animal) were captured with a digital camera (Axiocam MRc) connected to a microscope (AxioObserver, Carl Zeiss) using a 63x/1.25 immersion oil objective. The number of eosinophils in each image was determined and the total number of eosinophils per animal was divided by the total lung area analyzed. Results were expressed as the eosinophils/100mm^2^ +/- SD.

### Flow cytometry analysis

For cytometry analysis, lungs from PBS, Asthma or Smteg/Asthma were collected and treated with 100 U/mL of collagenase from *Clostridium histolyticum* (Sigma-Aldrich) for 30min at 37°C. Subsequently, the digested lung tissue was filtered through a 70 μm cell strainer and erythrocytes were lysed with a hemolytic solution (155 mM NH_4_Cl, 10 mM KHCO_3_, pH 7.2). The cell suspension was washed in RPMI 1640 (Gibco) and adjusted to 1x10^6^ cells/well. These cells were cultured overnight at 37°C in RPMI 1640 supplemented with 2 mM L-glutamine, 25 mM HEPES, 10% heat-inactivated FBS, penicillin G sodium (100 U/ml), and streptomycin sulfate (100 μg/ml). Brefeldin A (1μg/well, Sigma-Aldrich) was added 4 hrs before staining. Cells were then stained for surface and intracellular markers. Briefly, cells were incubated for 20 min with anti-mouse CD16/32 to block Fc receptors (eBioscience, San Diego, CA) in FACS buffer (PBS, 0.25% BSA, 1 mM NaN3) and were stained for surface markers for another 20 min. Next, Streptavidin was added. Cells were washed after 20 min, fixed in a 2% formaldehyde solution, and permeabilized with 0.5% saponin solution in PBS. After that, cells were stained for intracellular markers for 30 min. Then, cells were washed with permeabilization solution and ressuspended in PBS. The events were accquired using a LSRFortessa flow cytometer (BD), and data were analyzed using FlowJo Software (Tree Star, Ashland, OR, USA). The following molecules were used: FITC-conjugated anti-mouse CD4 (clone GK1.5, BD-Bioscience), FITC-conjugated anti-mouse CD11c (clone N418, eBioscience), biotin-conjugated anti-mouse CD3e (clone 500A2, BD-Bioscience), biotin-conjugated anti-mouse F4/80 (clone BM8, eBioscience), APC-Cy7-conjugated anti-mouse CD11b (clone M1/70, BD), eFluor 450-conjugated anti-mouse IFN-γ (clone XMG1.2, eBioscience), APC-conjugated anti-mouse IL-10 (clone JES5-16E3, BD-Bioscience), PE-conjugated anti-mouse Foxp3 (clone NRRF-30, eBioscience), Streptavidin APC-Cy7 (BD-Bioscience) and Streptavidin PerCP (BD-Bioscience).

### Statistical analysis

Statistical analysis was performed following Kolmogorov-Smirnov test to verify if the values have a Gaussian distribution. Next, it was performed Student's t test, One-way or Two-way ANOVA test using computer software GraphPad Prism 4 (GraphPad Software, San Diego, CA, USA).

### Ethics

All procedures involving animals were approved by the local Ethics Commission on Animal Use (CEUA) from Fiocruz (Protocol #LW39-10).

## Results

### Smteg treatment reduces airway inflammation

During airway inflammation, high levels of proteins are detected in BAL, an evidence for tissue damage. Moreover, inflammatory cellular infiltrate is characteristic of this condition. The comparison between PSB and Asthma groups showed that airway inflammation was successful induced (S1 Fig).

**Fig 2:**
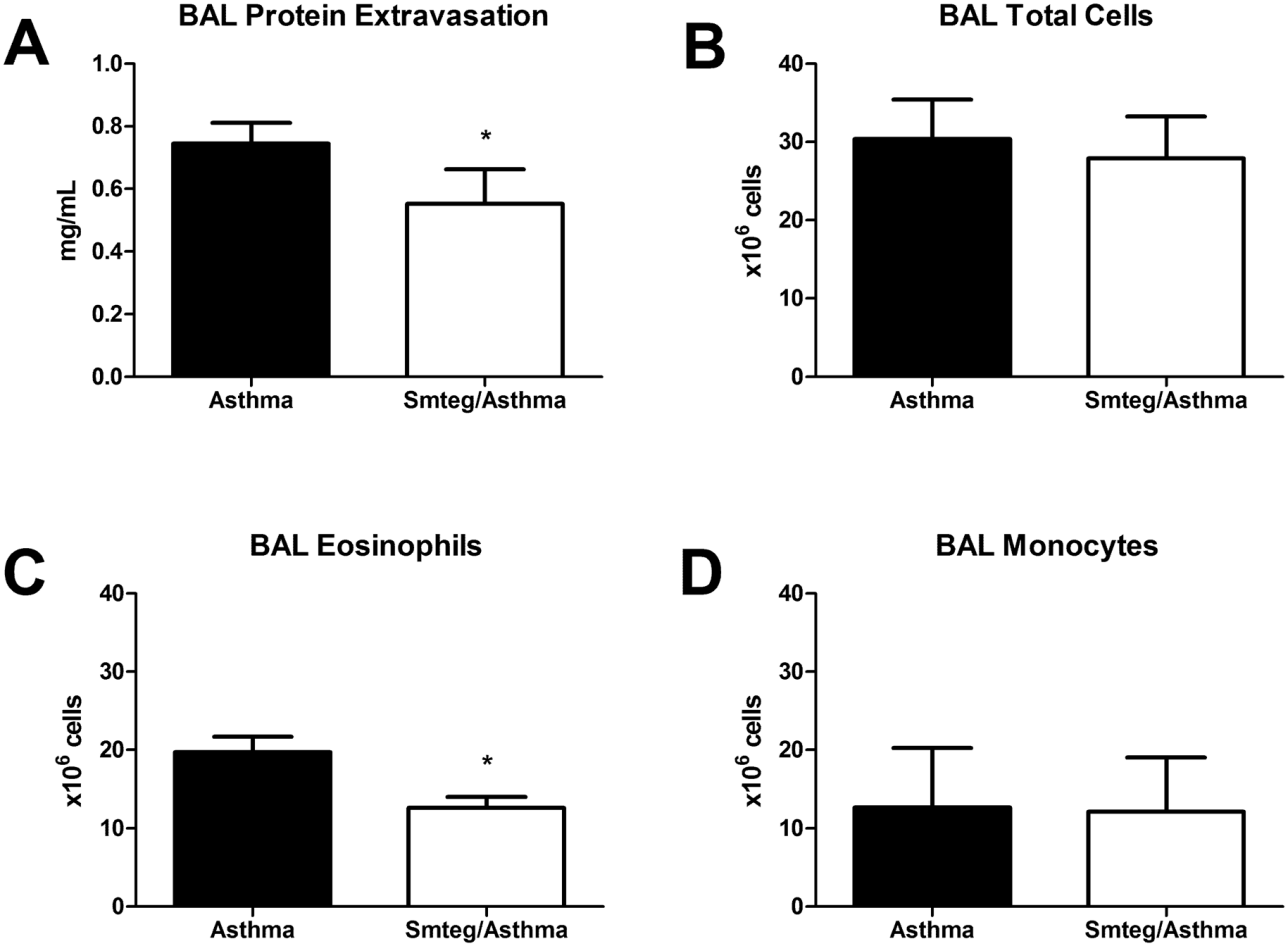
Reduction in airway inflammation induced by Smteg treatment. (**A**) Proteins extravasation, (**B**) the numbers of total cells, (**C**) eosinophils and (**D**) monocytes were quantified in BAL. The treatment with Smteg reduced significantly the protein extravasation and eosinophil counting compared to Asthma group. *p < 0,05; Student's t test. Data are representative of 2 independent experiments. Results are presented as mean ± SD.

In this mice model, Smteg treatment led to significant reduction in protein extravasation and eosinophils in BAL (Fig 2A and 2C). The numbers of total cells and monocytes did not significantly change in the Smteg/Asthma group compared to Asthma group (Fig 2B and 2D). Therefore, the Smteg treatment tested modulates airway inflammation.

### Smteg treatment enhances IL-10 production whereas decreases proinflammatory cytokines and IgE levels

**Fig 3:**
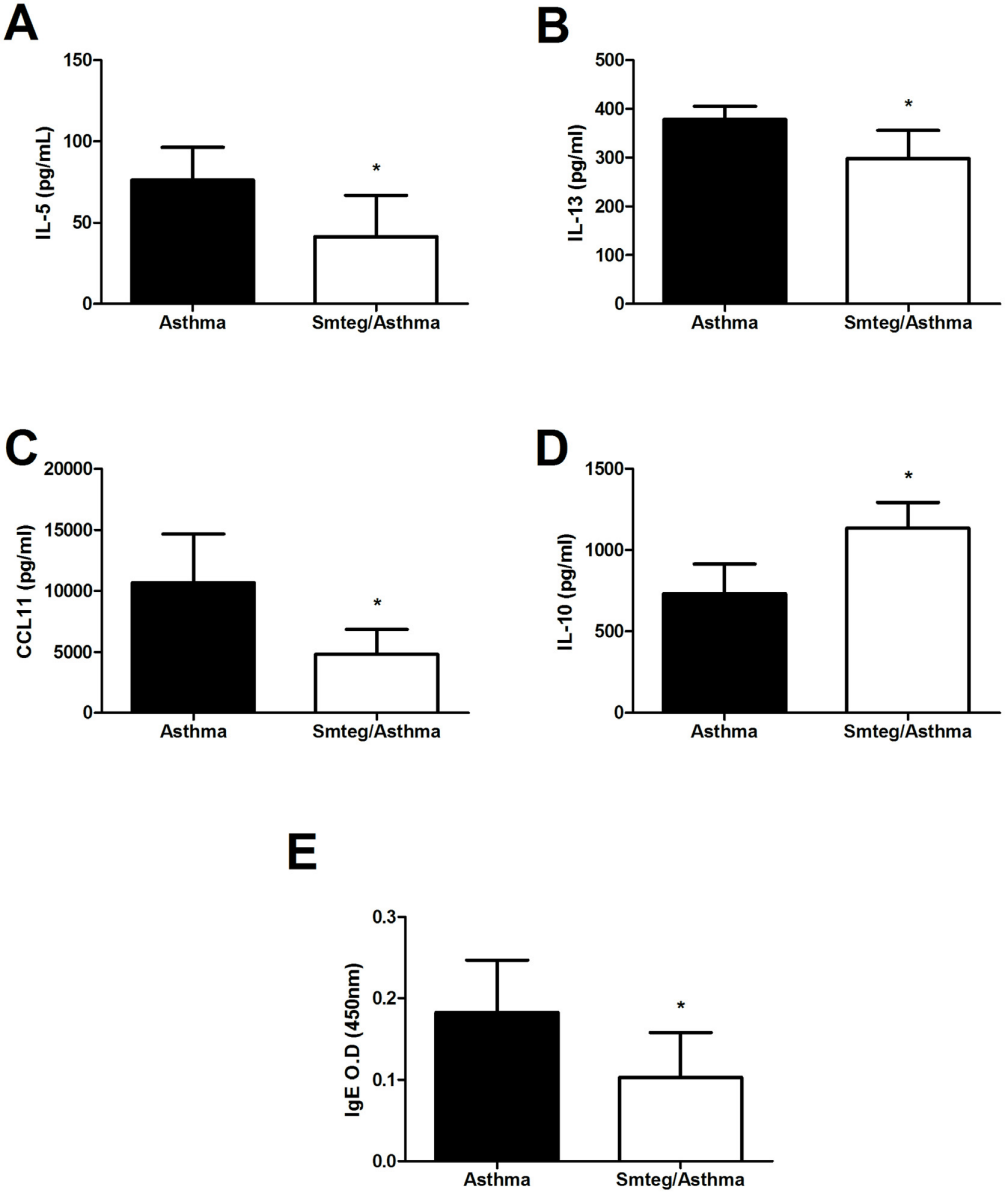
Smteg treatment reduced inflammatory parameters of airway inflammation. The production of (**A**) IL-5, (**B**) IL-13, (**C**) CCL11 and (**D**) IL-10 was evaluated in the lungs of mice. The Smteg/Asthma group presented lower levels of inflammatory cytokines (A-C) while showed up-regulation of IL-10 (D), compared to Asthma group. Moreover, (**E**) OVA-specific IgE levels in sera of mice were reduced due to Smteg treatment. *p < 0,05; Student's t test; O.D = Optical Density. Data are representative of 2 independent experiments. Results are presented as mean ± SD.

In order to evaluate the immunological microenvironment in lungs, the tissue was collected and analyzed for the presence of several important cytokines in allergy. As shown in Fig 3, there was a significant reduction in the proinflammatory cytokines IL-5, IL-13 and CCL11 while a significant increase in IL-10 levels was observed. Moreover, it was observed a reduction in anti-OVA IgE titers in blood samples from mice of the Smteg/Asthma group, compared to the Asthma group. Smteg injection per se led to an increased production of IL-10 by spleen cells without inducing the production of important proinflammatory cytokines such as IL-4, IFN-γ or IL-17 (S2 Fig), suggesting an important role of tegument’s molecules in immunomodulation.

### Reduced lung pathology in Smteg treated mice

**Fig 4:**
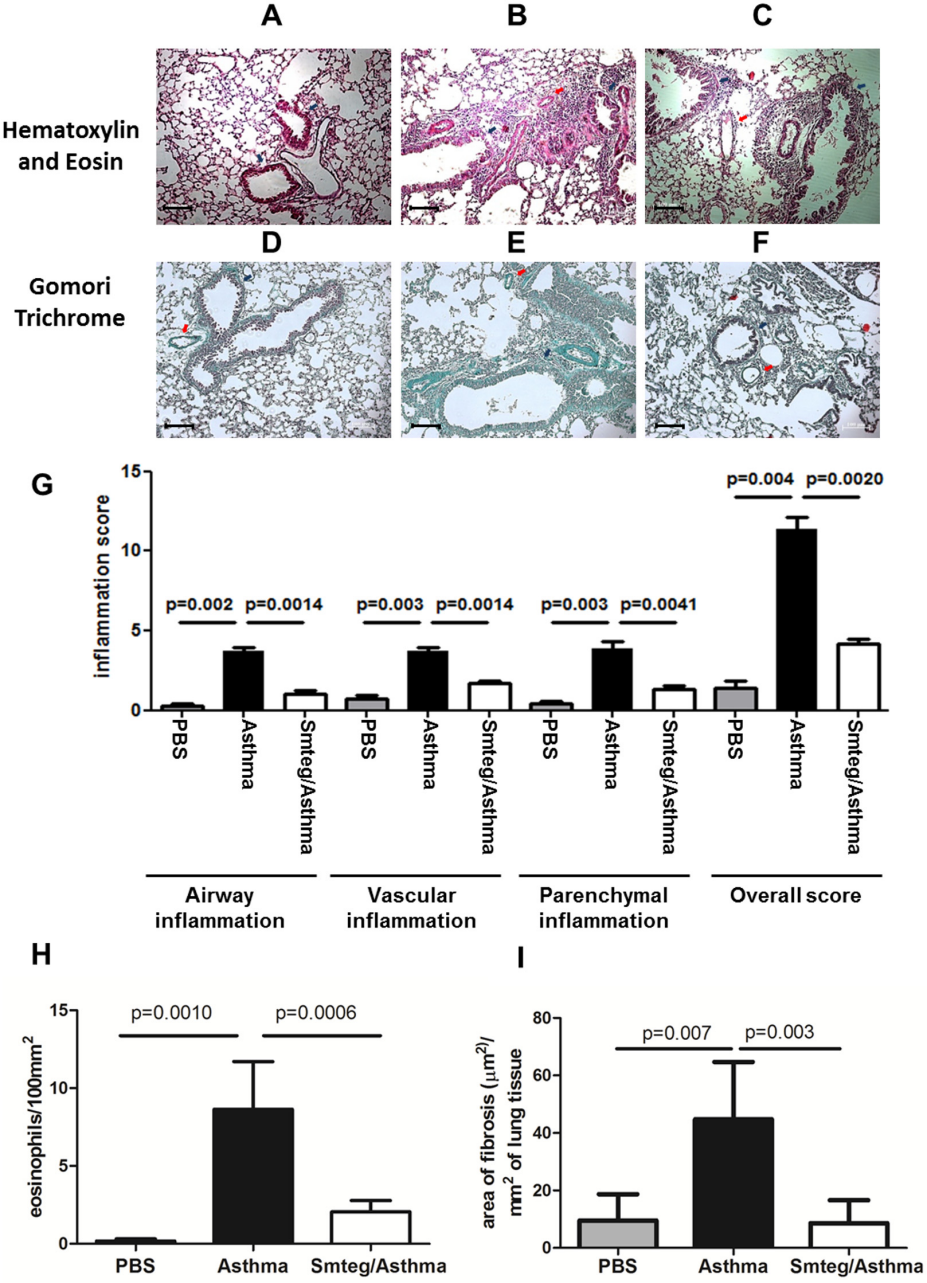
Lungs histopathology analysis. Upper panel shows lungs stained with HE and bottom panel shows lungs stained with Gomori Trichrome. Lungs from PBS control group (**A** and **D**) presented almost no infiltrate and collagen deposition. Asthmatic mice (**B** and **E**) presented intense inflammatory infiltrate and collagen deposit. Animals treated with Smteg (**C** and **F**) showed reduced levels of these parameters. Blue arrows point to peribronchiolar area and red arrows point to perivascullar area. Magnification 10x; bars 100μm. Semiquantitative analysis of inflammation (**G**) was performed in lung sections from five animals per group in magnification of 20x. Increased inflammation was observed in Asthma group compared to PBS and Smteg treated mice. Eosinophil number was determined in lung section (**H**) from five animals per group at a magnification of 63x. Results were expressed as the mean number of eosinophil/100mm^2^ +/- SD. Smteg treatment significantly decreased the number of eosinophils in the lung. Collagen deposition was measured by morphometric analysis of lung sections stained with Gomori Trichrome (I) at a magnification of 10x. Results are expressed as area of fibrosis (μm^2^)/mm^2^ of lung tissue. Significant reduction in collagen deposition was observed in Smteg treated group. Significant differences between groups are pointed in the Graphics. Data are representative of 2 independent experiments.

Histological sections of lungs from mice were stained with HE (Fig 4) and used to evaluate the inflammatory cell infiltrate. Also, a staining with Gomori Trichrome was used to analyze collagen deposit in lungs (Fig 4). An exacerbated inflammatory response, characterized by an intense presence of inflammatory cells was observed in lungs of mice from the Asthma group (Fig 4B, upper panel) in comparison to PBS control group (Fig 4A, upper panel). Markedly, a reduction in this pulmonary inflammation was observed in mice from the Smteg/Asthma group (Fig 4C upper panel) compared to mice from Asthma group. Semiquantitative analysis indicate that Smteg treatment decrease significantly perivascular, airway and parechymal inflammation (Fig 4G) Also a decreased number of eosinophil was observed in lung tissue of animals from the Smteg/Asthma group compared to mice from asthma group (Fig 4H). Concerning collagen, great deposition in perivascullar (red arrows) and peribronchiolar (blue arrows) areas was observed at lungs in mice from the Asthma group (Fig 4E and 4I) comparing to PBS group (Fig 4D and 4I). This collagen deposition was reduced in Smteg treated mice (Fig 4F and 4I)

### Increased presence of IL-10 producing monocytes in lungs of Smteg treated mice

**Fig 5:**
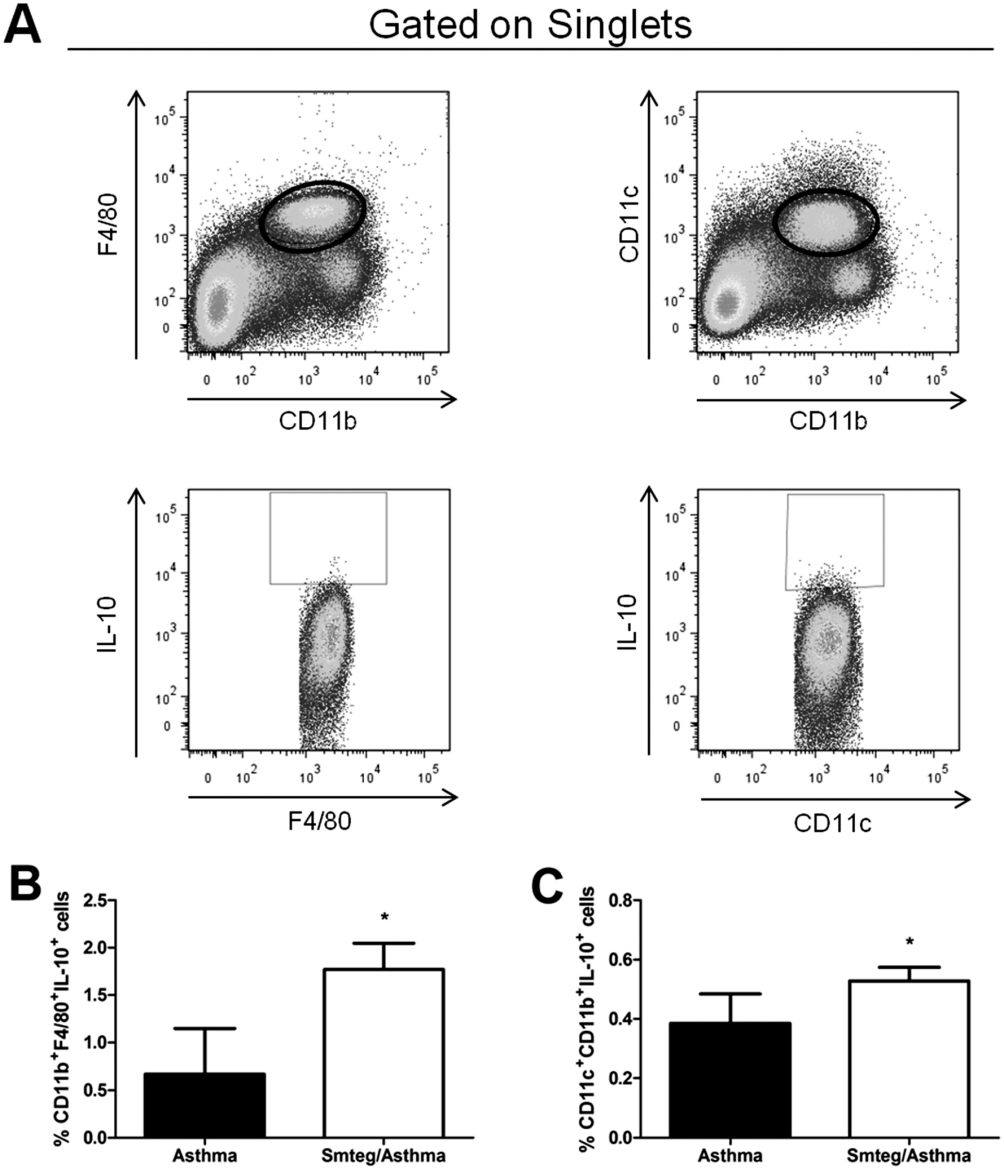
Increased production of IL-10 by monocytes in lungs of Smteg treated mice. Lungs cells were stained as described in Materials and Methods. Analysis strategy was represented in **A**. Mice of the Smteg/Asthma group presented higher percentage of IL-10 producing cells expressing (**B**) macrophage or (**C**) dendritic cells markers compared to Asthma group. *p < 0,05; Student's t test.

The cytokine IL-10 can be produced by several cell types. To investigate the source of this cytokine in lungs of mice with airway inflammation induced by OVA, flow cytometry was performed. The Smteg treated mice presented significant increased percentage of CD11b^+^F4/80^+^IL-10^+^ and CD11c^+^CD11b^+^IL-10^+^ cells compared to Asthma group (Fig 5). Moreover there were no differences concerning CD3^+^CD4^+^Foxp3^+^, CD3^+^CD4^+^IL-10^+^, CD3^+^CD4^+^IFN-γ^+^, CD3^+^CD4^+^IL-10^+^IFN-γ^+^ or CD3^-^CD19^+^IL-10^+^ cells between Asthma and Smteg/Asthma groups (S3 Fig). This result suggests an important role of IL-10 producing monocytes in regulating inflammation in the lung of Smteg treated mice.

## Discussion

Allergic asthma is an inflammatory airways disease which prevalence is lower in endemic areas for schistosomiasis [11, 13]. This negative correlation was reproduced in murine model and had been associated to some schistosomiasis induced features such as IL-10 and TGF-β production and increased numbers of regulatory T and B cells [21, 29–30]. Recently, our group demonstrated that Smteg was able to induce IL-10 production by DCs and by CD4^+^ cells [25]. Herein, we investigated the ability of Smteg injected intraperitoneally to modulate the experimental airway inflammation induced by ovalbumin.

One of the most important feature of allergic asthma is the lung tissue inflammation where the eosinophils are important cells for production of proinflammatory mediators that exacerbate the inflammatory response against allergens [31–32]. This pathological process can be indirectly measured by analysis of protein extravasation observed in BAL. Smteg inoculation reduced markedly both protein extravasation and eosinophils presence in BAL, without influencing significantly monocytes or total cells counting (Fig 2). Additionally, its ability to modulate asthma was reinforced by the analysis of histological sections from lungs where asthmatic mice showed excessive inflammatory infiltrate, increased number of eosinophils and collagen deposition comparing to Smteg treated animals (Fig 4). Moreover, mice that received Smteg showed reduction in important inflammatory cytokines and chemokines in lungs, such as IL-5, IL-13 and CCL11 (Fig 3). IL-5 and CCL11 are important cytokines for the development, expansion and mobilization of eosinophils from bone marrow, which can leads to eosinophilic infiltration following antigen exposure [33–35]. The downregulation of these molecules may be related to the low eosinophils recruitment after asthma induction in Smteg inoculated mice. Regarding IL-13, one of its marked characteristics is contribution to asthma pathogenesis through goblet cell hyperplasia and collagen deposition [36], which was reduced in Smteg treated mice (Figs 3B and 4). The data indicating reduction in all these pro-inflammatory cytokines was accompanied by increased IL-10 levels in lungs of Smteg inoculated mice (Fig 3E). This cytokine have a central role in down-regulating inflammatory responses. Moreover, besides the modulatory environment, it was observed reduction in anti-OVA IgE levels in sera of mice treated with Smteg (Fig 3F). It is important to note that inoculation of Smteg per se increased IL-10 levels without influencing IL-4, IFN-γ or IL-17A levels in naïve mice (S2 Fig). The investigation to identify the source of IL-10 in this model revealed increased percentage of CD11b^+^F4/80^+^IL-10^+^ and CD11c^+^CD11b^+^IL-10^+^ cells in Smteg treated mice (Fig 5). Nevertheless, there were no difference in CD3^+^CD4^+^Foxp3^+^, CD4^+^IL-10^+^ or CD4^+^IFN-γ^+^IL-10^+^ cells between Asthma and Smteg/Asthma groups (S3 Fig). In addition, there was no difference in CD4^+^IFN-γ^+^ or CD3^-^CD19^+^IL-10^+^ population between these groups (S3 Fig). These data suggest that macrophages and dendritic cells are the main source of IL-10 in lungs of Smteg inoculated mice, being responsible for the modulatory environment in the animal model presented here. It is noteworthy the role of innate over adaptive immunity in Smteg treated airway inflammation.

The results presented here demonstrated that Smteg inoculation was able to reduce pro-inflammatory cytokines and IgE levels while enhanced IL-10 production by monocytes. The balance of these immune mediators is an important parameter to be evaluated in airway inflammatory disorders. IL-10 is an anti-inflammatory cytokine, what renders it a promising molecule for therapeutic intervention [37]. During *S*. *mansoni* infection, the transition from acute to chronic phase is marked by increased levels of IL-10 [38]. This cytokine have an important role in controlling morbidity of schitosomiasis, contributing to host survival as well as down-regulating immune response to parasite [39]. High levels of IL-10 induced by Smteg inoculation shed light to a possible mechanism of modulation induced by schistosomula tegument in allergic asthma. However, it is worth mentioning that this study proposes a role for Smteg in preventing allergy, as its administration occurs before the challenge. The results suggest that the immunomodulatory environment established by Smteg injection is strong enough to prevent the development of the inflammatory allergic reaction. Moreover, the data presented here corroborates with a previous study that demonstrated a significant production of IL-10 by bone-marrow derived dendritic cells stimulated with Smteg [25].

The IL-10 augmentation could explain the reduced levels of IgE in sera from mice that received Smteg. In humans, IL-10 potentiates IgG4 production and decreases IgE synthesis [40], an action important for asthma modulation. A genetic study reinforce the opposite relation between IgE and IL-10, showing that polymorphisms in IL-10 promoter were associated with high total serum IgE and increased risk for asthma [41]. IgE is a hallmark of allergic disease. The antigen-dependent cross linking of IgE on mast cell surface leads to its degranulation and inflammatory mediators release, that will promote the asthma symptomatology as mucus hypersecretion, airway obstruction and hyperresponsiveness, breathlessness and coughing [42]. The low levels of IgE observed in this work is in agreement with previous studies that demonstrated an association between low levels of specific anti-OVA IgE and modulation of induced airway inflammation in murine models [18, 21, 29, 43].

In conclusion, this study demonstrates that Smteg treatment in an experimental model of airway inflammation induced by OVA reduces eosinophils in BAL and lung tissue, as well as tissue damage, specific anti-OVA IgE and IL-5, IL-13, IL-25 and CCL11 levels in lungs, diminishing overall airway pathology. One possible mechanism involved in this modulation is the production of the regulatory cytokine IL-10 by macrophages and dendritic cells, that was increased in lungs in this experimental model. This work expands the knowledge of schistosomula tegument properties and its modulatory effect can be used by the lung stage parasite to evade host immune responses. Furthermore, this study presents a rational to use parasite compounds to a therapeutic intervention. More studies are necessary to elucidate the complete modulatory mechanism induced by Smteg and determine the crucial molecules involved in this process.

## Supporting Information

S1 Fig Induction of airway inflammation successfully performed. Mice were submitted to the protocol described in Materials and Methods. (**A**) Numbers of total cells, (**B**) eosinophils and (**C**) monocytes were evaluated in BAL. The Asthma group presented increased counting of cells. *p < 0,05 compared to PBS; Student's t test. Data are representative of 2 independent experiments. Results are presented as mean±SD.
(TIF)

S2 Fig Smteg induces production of IL-10. Mice were treated with PBS or Smteg alone and spleen cells supernatant were analyzed for (**A**) IL-4, (**B**) IFN-γ, (**C**) IL-17A and (**D**) IL-10. Smteg treatment induced IL-10 production compared to PBS mice. *p < 0,05 compare do PBS; Two Way ANOVA followed by Bonferroni post-test.
(TIF)

S3 Fig Smteg treatment did not increased regulatory lymphocytes. Lungs cells were stained as described in Materials and Methods. Analysis strategies were represented in **A** and **B**. There were no difference in (**C**) CD3^+^CD4^+^Foxp3^+^ cells, (**D**) CD3^+^CD4^+^IL-10^+^, (**E**) CD3^+^CD4^+^IFN-γ^+^, (**F**) CD4^+^IL-10^+^IFN-γ^+^ or (**G**) CD3^-^CD19^+^IL-10^+^ comparing Asthma or Smteg/Asthma groups.
(TIF)
