# Supplementary figures and images for: Schistosoma mansoni Tegument (Smteg) Induces IL-10 and Modulates Experimental Airway Inflammation
Source: PLoS One. 2016 Jul 25;11(7):e0160118. doi: 10.1371/journal.pone.0160118 (PMC4959726; doi:10.1371/journal.pone.0160118)

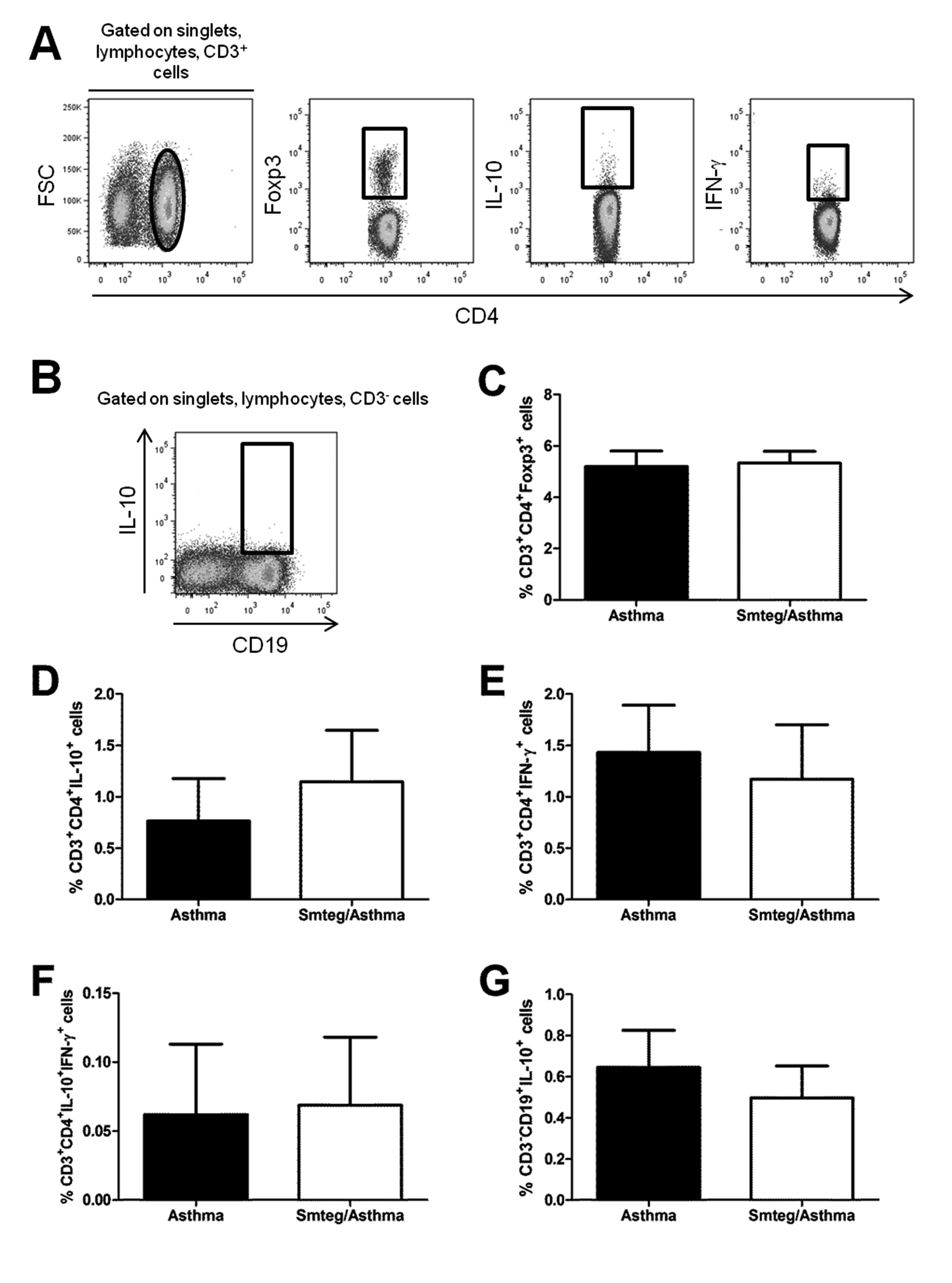

Supplement: S3 Fig — Lungs cells were stained as described in Materials and Methods. Analysis strategies were represented in A and B. There were no difference in (C) CD3+CD4+Foxp3+ cells, (D) CD3+CD4+IL-10+, (E) CD3+CD4+IFN-γ+, (F) CD4+IL-10+IFN-γ+ or (G) CD3-CD19+IL-10+ comparing Asthma or Smteg/Asthma groups. (TIF) [file pone.0160118.s003.tif]
